# Arbeitsbedingungen in der endokrinen Chirurgie: Eine Studie unter Teilnehmern der 43. Jahrestagung der Chirurgischen Arbeitsgemeinschaft Endokrinologie

**DOI:** 10.1007/s00104-026-02529-7

**Published:** 2026-06-17

**Authors:** Maximilian Kießler, Maximilian Berlet, Anna Mikl, Helmut Friess, Costanza Chiapponi

**Affiliations:** Klinik und Poliklinik für Chirurgie, Klinikum rechts der Isar, Technische Universität München, Ismaninger Str. 22, 81675 München, Deutschland

**Keywords:** Berufliche Zufriedenheit, Chirurgische Subspezialisierung, Weiterbildung, Burnout-Risiko, Psychische Gesundheit, Job satisfaction, Surgical subspecialization, Postgraduate training, Burnout risk, Psychiatric health

## Abstract

**Einleitung:**

Die Arbeitsbedingungen in chirurgischen Fachdisziplinen sind zunehmend durch hohe Belastung und wachsende Unzufriedenheit geprägt, was sich negativ auf Nachwuchsgewinnung, Berufsverbleib und langfristige Versorgungsstrukturen auswirkt. Während hierzu umfangreiche Daten für die Chirurgie insgesamt vorliegen, wurden die Arbeitsbedingungen in der endokrinen Chirurgie als Subspezialisierung der Viszeralchirurgie bislang nicht gesondert untersucht. Ziel der vorliegenden Studie war es, erstmals die berufliche Zufriedenheit, Belastungsfaktoren und Arbeitsbedingungen endokrin-chirurgisch tätiger Ärztinnen und Ärzte in Deutschland zu erfassen und diese in Abhängigkeit vom Umfang der endokrinen Tätigkeit zu vergleichen.

**Material und Methoden:**

Im Rahmen der 43. Jahrestagung der Chirurgischen Arbeitsgemeinschaft Endokrinologie (CAEK) wurde eine anonyme Querschnittsumfrage durchgeführt. Der standardisierte Fragebogen umfasste 28 Items zu soziodemografischen Daten, beruflicher Tätigkeit, Arbeitsbelastung und beruflicher Zufriedenheit.

**Ergebnisse:**

Insgesamt konnten 84 vollständig ausgefüllte Fragebögen in die Analyse eingeschlossen werden. Insgesamt berichteten 85 % der Befragten von einer *eher hohen* oder *sehr hohen* beruflichen Zufriedenheit. Eine überwiegende Tätigkeit in der endokrinen Chirurgie war mit einer signifikant höheren allgemeinen Zufriedenheit assoziiert (OR = 2,78; *p* = 0,021). Besonders ausgeprägt waren die Unterschiede in den Bereichen Fortbildungsmöglichkeiten (OR = 4,41; *p* = 0,001), Arbeitszeitregelung (OR = 3,21; *p* = 0,006) und Weiterbildung (OR = 2,93; *p* = 0,014); 75 % der Befragten gaben an, dass sie sich erneut uneingeschränkt für die endokrine Chirurgie entscheiden würden. Hauptbelastungsfaktoren waren hingegen bürokratische und organisatorische Anforderungen. Das Belastungsniveau unterschied sich insgesamt kaum zwischen den Gruppen. Lediglich die Belastung durch Nachtdienste war bei nachrangig endokrin-chirurgisch Tätigen signifikant höher.

**Schlussfolgerung:**

Ärztinnen und Ärzte mit überwiegender Tätigkeit in der endokrinen Chirurgie weisen trotz vergleichbarer Belastung ein signifikant höheres Maß an beruflicher Zufriedenheit auf. Die Ergebnisse legen nahe, dass insbesondere geeignete strukturelle und organisatorische Rahmenbedingungen eine zentrale Rolle für die Attraktivität dieses Fachgebiets spielen könnten. Die Schaffung entsprechender Voraussetzungen könnte dazu beitragen, die berufliche Zufriedenheit zu fördern und die Nachwuchsbindung langfristig zu stärken.

**Graphic abstract:**

**Figure:**
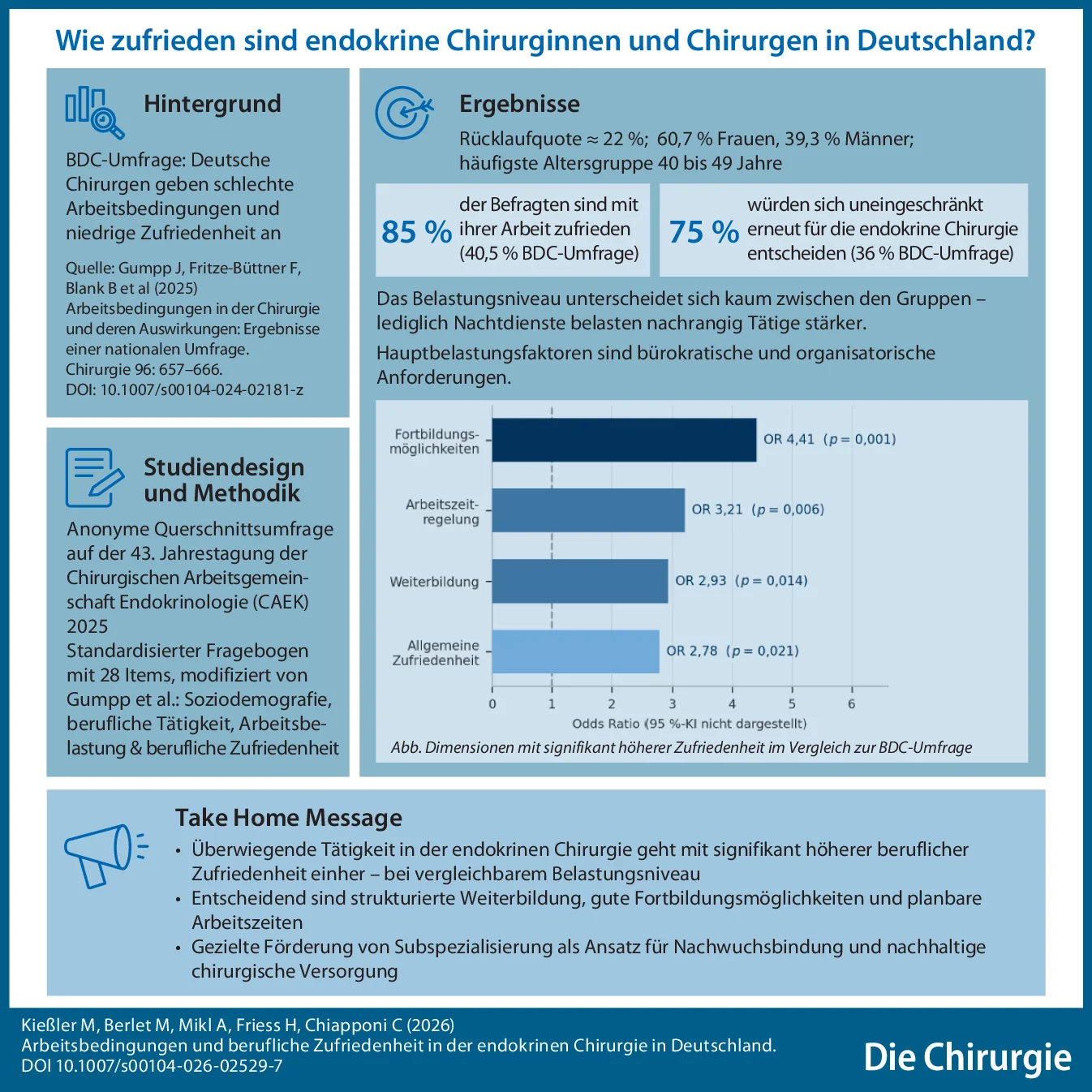

## Abstract (en)

**Introduction:**

Working conditions in surgical specialties are increasingly characterized by high workload and growing job dissatisfaction, negatively affecting recruitment, retention, and the long-term sustainability of healthcare services. While extensive data are available for surgery as a whole, working conditions in endocrine surgery as a subspecialty of visceral surgery have not yet been investigated separately. The aim of the present study was, for the first time, to assess job satisfaction, occupational stressors, and working conditions among endocrine surgeons in Germany and to compare these factors according to the extent of endocrine surgical practice.

**Materials and Methods:**

An anonymous cross-sectional survey was conducted during the 43rd Annual Meeting of the Surgical Working Group for Endocrinology (CAEK). The standardized questionnaire comprised 28 items addressing sociodemographic characteristics, professional activity, workload, and job satisfaction.

**Results:**

A total of 84 fully completed questionnaires were included in the analysis. Overall, 85% of respondents reported high or very high job satisfaction. Predominant involvement in endocrine surgery was associated with significantly higher overall job satisfaction (OR = 2.78; p = 0.021). Differences were particularly pronounced regarding opportunities for continuing medical education (OR = 4.41; p = 0.001), working time arrangements (OR = 3.21; p = 0.006), and surgical training and professional development (OR = 2.93; p = 0.014). Furthermore, 75% of respondents stated that they would unequivocally choose endocrine surgery again as their specialty. The main sources of occupational burden were bureaucratic and organizational demands. Overall stress levels differed only marginally between groups. The only significant difference was a higher burden from night shifts among surgeons whose endocrine surgical practice constituted a secondary part of their professional activity.

**Conclusion:**

Physicians predominantly engaged in endocrine surgery demonstrate significantly higher job satisfaction despite experiencing comparable levels of occupational burden. These findings suggest that specialized clinical profiles, structured training pathways, and favorable working time models contribute substantially to the attractiveness of this field. Promoting specialized training with the goal of subspecialization may therefore represent an important strategy for improving job satisfaction, enhancing recruitment and retention, and ensuring the long-term provision of surgical care in other areas of visceral surgery as well.

Die Arbeitsbedingungen in chirurgischen Fachdisziplinen stehen zunehmend in der Kritik. Viele Chirurginnen und Chirurgen berichten von zunehmender Arbeitsbelastung und wachsender Unzufriedenheit. Dies hat gravierende Folgen: Studien zeigen, dass sich die Belastungen im Berufsalltag negativ auf die psychische Gesundheit und das Familienleben auswirken und viele Ärztinnen und Ärzte in Erwägung ziehen, den Beruf frühzeitig aufzugeben. Eine aktuelle bundesweite Umfrage des Berufsverbands der Deutschen Chirurgie (BDC) aus dem Jahr 2024 mit 2221 Teilnehmenden zeichnet ein alarmierendes Bild: Bürokratie (84,4 %) und ein unzureichender Ausgleich von Überstunden (68,1 %) wurden von Chirurginnen und Chirurgen als häufigste Stressoren benannt. Fast die Hälfte der Befragten (49 %) hatte aufgrund der Arbeitslast sogar schon darüber nachgedacht, aus dem chirurgischen Beruf auszusteigen. Ebenso berichteten 60 % von negativen Auswirkungen des Berufs auf ihre Partnerschaft und 40 % auf die Beziehung zu ihren Kindern [1].

Diese Daten sind vor dem Hintergrund eines zunehmenden Fachkräftemangels in der Medizin besonders brisant. Ein knappes Viertel des Klinikpersonals in Deutschland ist aktuell 55 Jahre oder älter, sodass in den nächsten Jahren viele erfahrene Kräfte altersbedingt ausscheiden werden [2]. Gleichzeitig ist die Zahl chirurgischer Eingriffe in Deutschland in den letzten 2 Jahrzehnten drastisch gestiegen [3]. Demgegenüber ist insbesondere bei Schilddrüsenoperationen in den letzten Jahren ein rückläufiger Trend zu beobachten [3], der potenziell Auswirkungen auf Ausbildungsstrukturen und die langfristige Entwicklung der endokrinen Chirurgie haben könnte.

Auch die Nachwuchsgewinnung gestaltet sich zunehmend schwieriger. Einer BDC-Umfrage zufolge ziehen nur noch 18 % der Medizinstudierenden im praktischen Jahr eine Karriere in einer chirurgischen Fachrichtung in Betracht [4]. Zwischen Studienbeginn und Examen sinkt der Anteil derjenigen mit dem Berufsziel Chirurgie drastisch – mehr als ein Drittel der Absolvierenden schließt eine chirurgische Laufbahn sogar gänzlich aus. Diese Entwicklungen verschärfen den Fachkräftemangel weiter und gefährden langfristig die Versorgung im chirurgischen Bereich [4].

Die endokrine Chirurgie stellt in diesem Kontext ein spezielles, bislang kaum gesondert untersuchtes Teilgebiet der Chirurgie dar. Als Subspezialisierung der Viszeralchirurgie fokussiert sie sich auf operative Eingriffe an hormonproduzierenden Organen (z. B. der Schilddrüse, den Nebenschilddrüsen und den Nebennieren) und weist zugleich charakteristische strukturelle Besonderheiten auf. Hierzu zählen ein hoher Anteil elektiver Eingriffe sowie eine vergleichsweise geringe Anzahl an Notfällen. Diese Merkmale könnten hypothetisch nicht nur mit spezifisch reduzierten Belastungsprofilen, sondern auch mit einer besonderen Attraktivität der Tätigkeit und einer höheren beruflichen Zufriedenheit einhergehen. Ob und in welchem Ausmaß sich die Arbeitsbedingungen, die berufliche Zufriedenheit und die Beanspruchung endokrin-chirurgisch tätiger Ärztinnen und Ärzte von denen der Chirurgie insgesamt (insbesondere der Viszeralchirurgie) unterscheiden, ist bislang jedoch unklar. Ziel der vorliegenden Studie war es daher, erstmals systematisch Einblicke in die Arbeitsbedingungen, Belastungsfaktoren und die berufliche Zufriedenheit von in der endokrinen Chirurgie tätigen Ärztinnen und Ärzten in Deutschland zu gewinnen. Ein besonderer Fokus lag dabei auf der Frage, ob eine überwiegende Tätigkeit in der endokrinen Chirurgie mit Unterschieden in Zufriedenheit und Belastung assoziiert ist.

## Material und Methoden

### Studiendesign und Datenerhebung

Die vorliegende Untersuchung basiert auf einer anonymen, querschnittlichen Online-Umfrage, die im Rahmen der 43. Arbeitstagung der Chirurgischen Arbeitsgemeinschaft Endokrinologie (CAEK) 2025 durchgeführt wurde. Die Kongressteilnehmenden wurden im Rahmen von Vorträgen vor Ort auf die Studie aufmerksam gemacht. Zusätzlich wurde eine Einladung zur Teilnahme per E-Mail versendet.

Der Fragebogen umfasste 28 Items und erhob soziodemografische Angaben, Informationen zur beruflichen Tätigkeit, zur Arbeitsbelastung sowie zur beruflichen Zufriedenheit.

Die Teilnahme war freiwillig und anonym. Es wurden keine personenbezogenen Daten erhoben.

### Statistische Analyse

Die Zufriedenheits- und Belastungsvariablen wurden auf einer 5‑stufigen Likert-Skala erhoben und als ordinale Variablen analysiert. Gruppenunterschiede wurden mittels ordinaler logistischer Regression (Proportional-Odds-Modell) untersucht. Für jedes Outcome wurde ein separates Modell mit der jeweiligen Gruppierungsvariable als Prädiktor berechnet.

Für die zweiseitige Hypothesentestung wurde ein Signifikanzniveau von 0,05 festgelegt. Alle Analysen wurden mit R (RStudio integrated development environment, Version 2025.12.1, Posit Software, PBC, Boston, MA, USA) durchgeführt [5].

## Ergebnisse

### Teilnehmer

Von insgesamt 385 Kongressteilnehmenden haben 84 (21,8 %) den Fragebogen vollständig ausgefüllt, davon 33 Männer (39,3 %) und 51 Frauen (60,7 %). Die Altersverteilung zeigte sich wie folgt: 1,2 % 20–29, 17,9 % 30–39, 44,0 % 40–49 und 23,8 % 50–60 und 13,1 % > 60.

Es gaben 83 % an in einer festen Partnerschaft/Ehe zu leben, 6,0 % gaben an, geschieden zu sein; 10,7 % gaben an, zum Zeitpunkt der Umfrage ledig zu sein. Von den in Partnerschaft lebenden Umfrageteilnehmenden gaben 39,7 % an, mit einem Partner mit ebenfalls medizinischem Hintergrund zusammen zu sein. Der überwiegende Teil der Befragten gab an, mindestens ein Kind zu haben (79,8 %), davon gaben 20,2 % der Befragten an, ein Kind, 38,1 % 2 Kinder, 14,3 % 3 Kinder und 7,1 % 4 oder mehr Kinder zu haben.

**Abb. 1:**
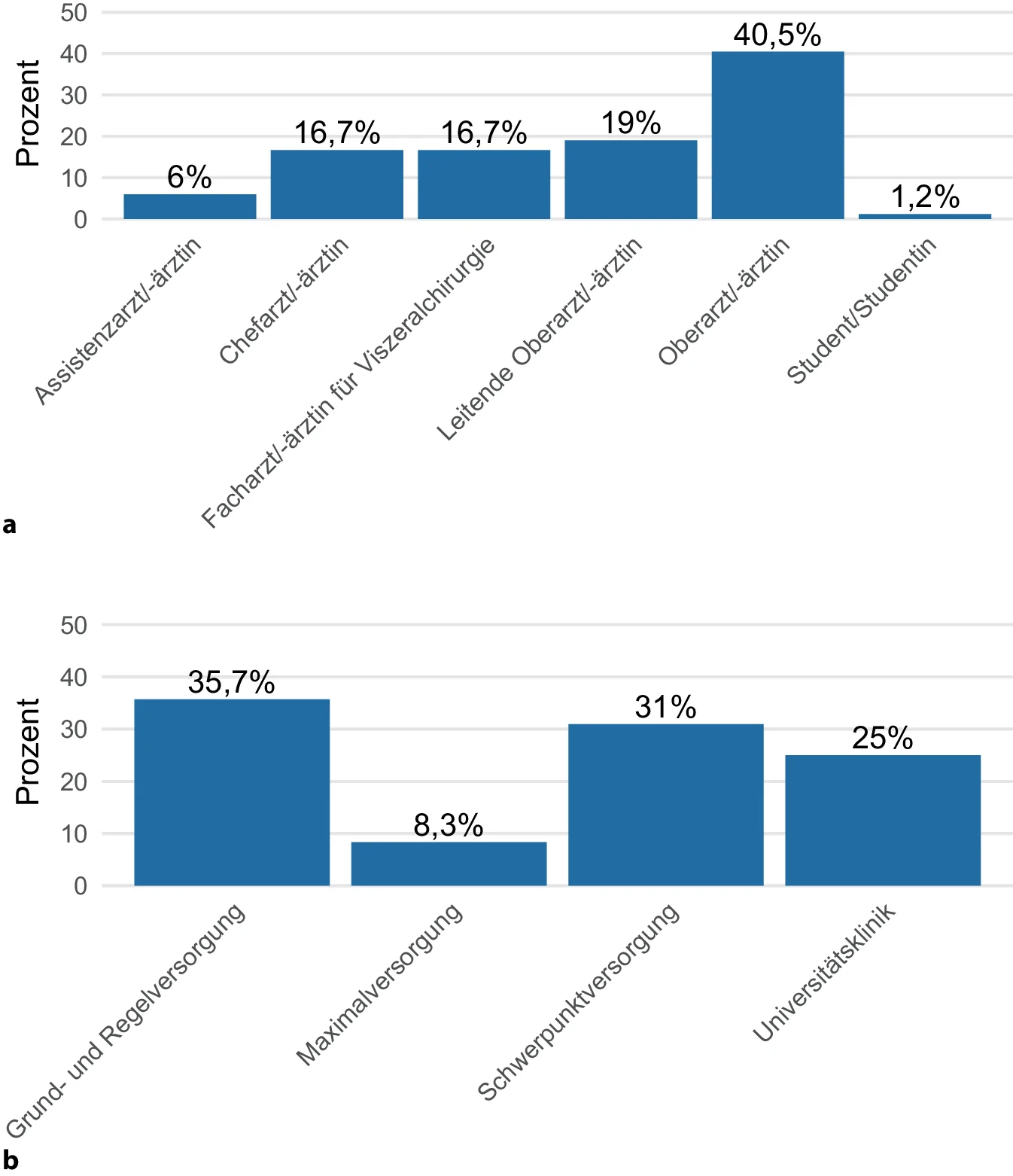
**a** Verteilung der Funktionsstellung unter den Befragten. **b** Krankenhaustypen, an denen die Befragten beschäftigt waren

Von den Befragten gaben 96,4 % an, aktuell aktiv in der endokrinen Chirurgie zu arbeiten, 3,6 % befanden sich zum Zeitpunkt der Umfrage im Krankenstand, in Elternzeit oder im Mutterschutz. Ein großer Teil (35,7 %) gab an, in einem Haus der Grund- und Regelversorgung tätig zu sein, gefolgt von Krankenhäusern der Schwerpunktversorgung (31,0 %), Universitätskliniken (25,0 %) und Krankenhäusern der Maximalversorgung (8,3 %) (Abb. 1).

Der mit Abstand größte Teil der Befragten gab an, als Oberarzt/-ärztin (40,5 %) tätig zu sein, gefolgt von leitendem Oberarzt/-ärztin (19,0 %), Facharzt/-ärztin und Chefarzt/-ärztin (je 16,7 %). Lediglich 6 % der Befragten gaben an, Assistenzarzt/-ärztin zu sein. Nur 1,2 % gaben an, Student/Studentin zu sein (s. Abb. 1). Zum Beschäftigungsumfang gaben 64,7 % an, in Vollzeit beschäftigt zu sein, gefolgt von 80 %-Teilzeit (25,0 % der Teilnehmenden) und 60 %-Teilzeit (9,4 % der Teilnehmenden).

Auf die Frage nach dem Umfang der Tätigkeit in der endokrinen Chirurgie antworteten 26,2 % der Befragten, ausschließlich in der endokrinen Chirurgie tätig zu sein, 22,6 % waren großteils in der endokrinen Chirurgie beschäftigt, 45,2 % waren teils in der endokrinen Chirurgie beschäftigt, 3,6 % waren gelegentlich in der endokrinen Chirurgie beschäftigt, und 2,4 % waren nur selten endokrin chirurgisch tätig.

### Zufriedenheit

**Abb. 2:**
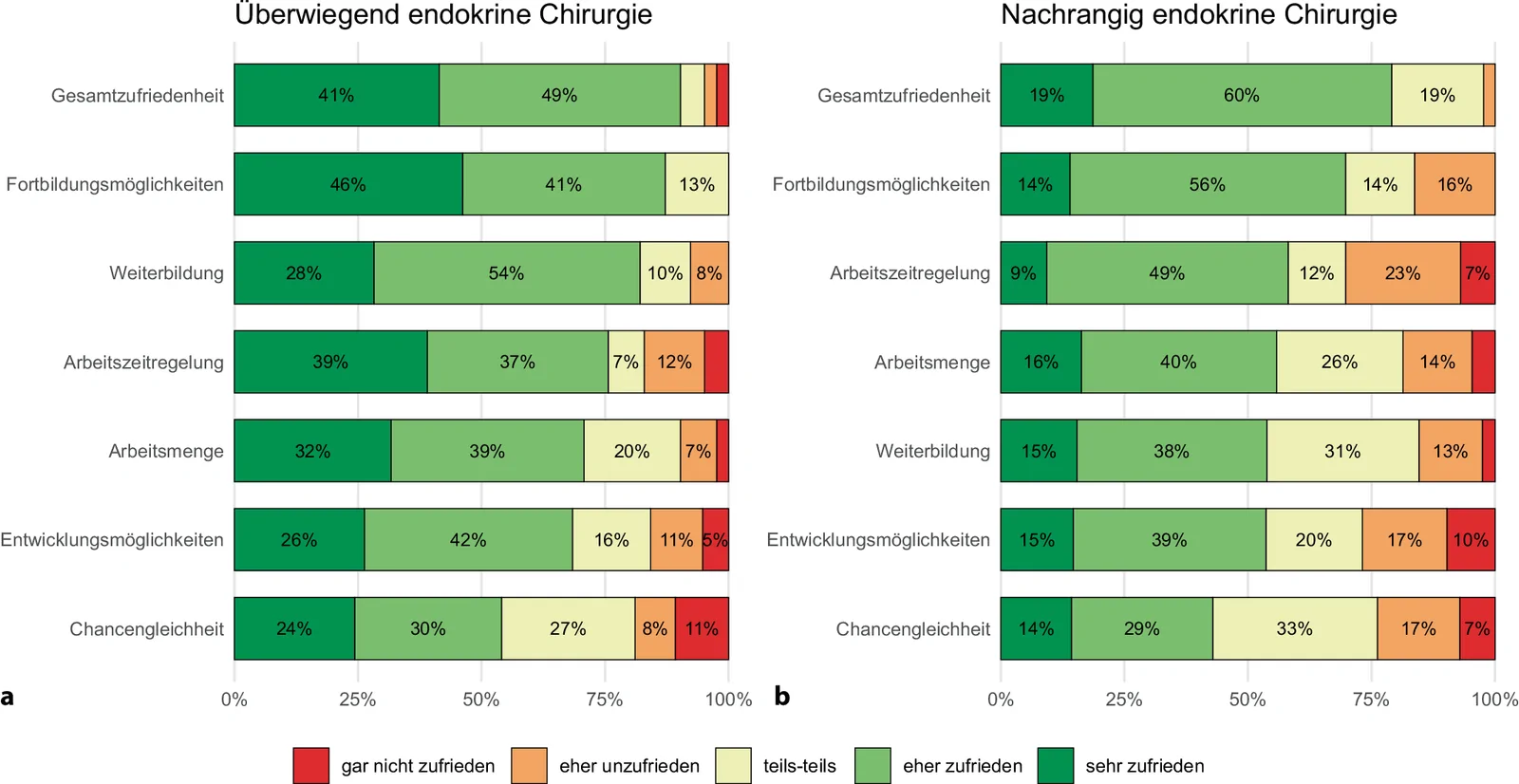
Berufliche Zufriedenheit in Abhängigkeit vom Umfang der Tätigkeit in der endokrinen Chirurgie. Dargestellt sind die prozentualen Anteile der Antwortkategorien (5-stufige Likert-Skala von „gar nicht zufrieden“ bis „sehr zufrieden“) für verschiedene Zufriedenheitsdimensionen. **a** Überwiegend endokrine Chirurgie (ausschließlich/großteils). **b** Nachrangig endokrine Chirurgie. Insgesamt berichteten 85 % der Befragten eine eher hohe oder sehr hohe Zufriedenheit

Die überwiegende Mehrheit (85 %) zeigte sich mit ihrer Arbeit zufrieden. Dabei war eine überwiegende Tätigkeit in der endokrinen Chirurgie (ausschließlich endokrine Chirurgie/großteils endokrine Chirurgie) mit einer signifikant höheren allgemeinen beruflichen Zufriedenheit assoziiert (OR = 2,78, *p* = 0,021). Entscheidende Faktoren für die höhere berufliche Zufriedenheit bei überwiegend in der endokrinen Chirurgie tätigen Ärztinnen und Ärzten waren insbesondere die Fortbildungsmöglichkeiten (OR = 4,41, *p* = 0,001), die Arbeitszeitregelung (OR = 3,21, *p* = 0,006) sowie die Weiterbildung (OR = 2,93, *p* = 0,014) (Abb. 2).

Es zeigte sich kein signifikanter Unterschied in der allgemeinen beruflichen Zufriedenheit zwischen Frauen und Männern.

Die Frage, ob die Teilnehmenden erneut in der endokrinen Chirurgie tätig werden würden, beantworteten 75 % uneingeschränkt mit („Ja, auf jeden Fall“), während 25 % angaben, dass sie dies unter veränderten Bedingungen erneut in Betracht ziehen würden. Keine der befragten Personen lehnte eine erneute Tätigkeit in der endokrinen Chirurgie ab.

### Belastung

**Abb. 3:**
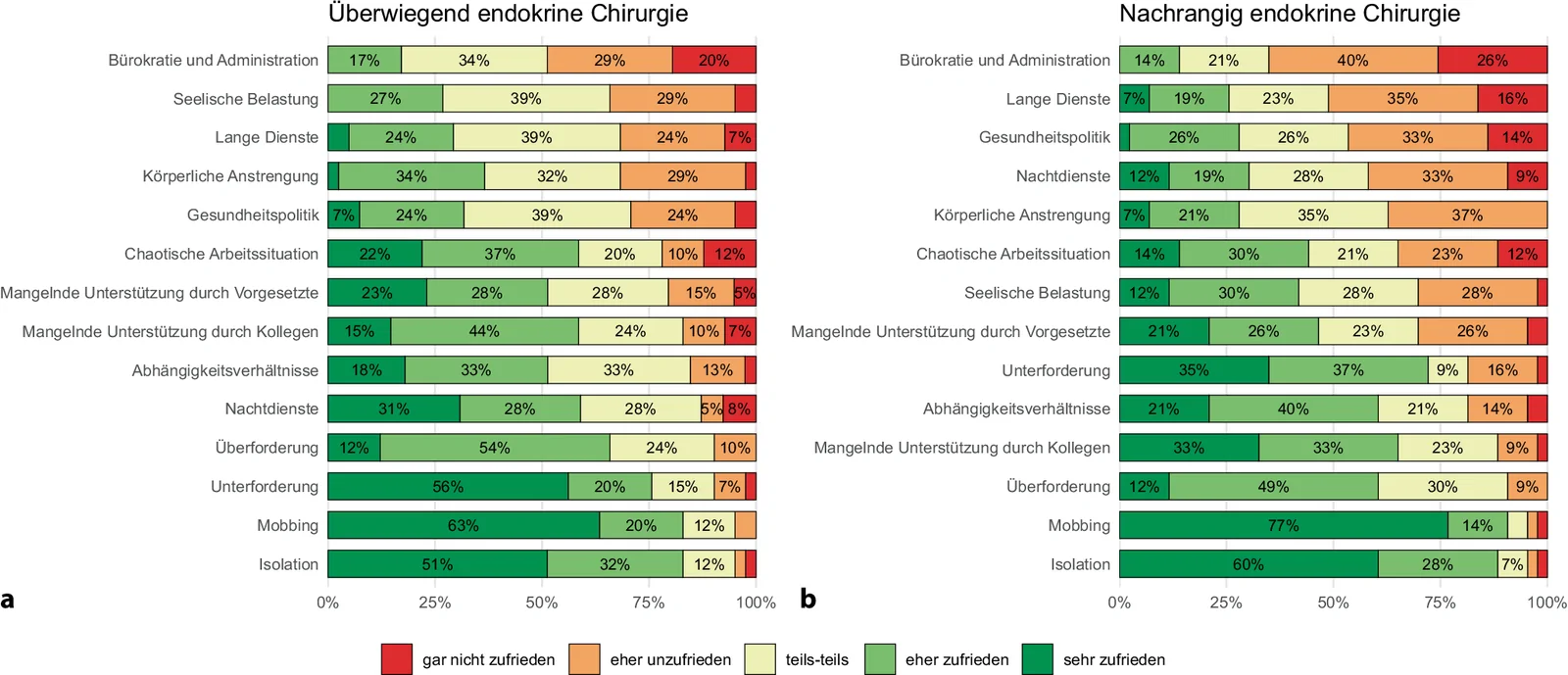
Berufliche Belastungsfaktoren in Abhängigkeit vom Umfang der Tätigkeit in der endokrinen Chirurgie. Dargestellt sind die prozentualen Anteile der Antwortkategorien (5-stufige Likert-Skala von „gar nicht belastet“ bis „stark belastet“). **a** Überwiegend endokrine Chirurgie. **b** Nachrangig endokrine Chirurgie

In der Gesamtstichprobe zeigte sich eine ausgeprägte Belastung durch strukturelle und organisatorische Faktoren. Besonders häufig wurden Bürokratie und Administration als belastend wahrgenommen: 58 % der Befragten gaben an, hierdurch häufig oder stark belastet zu sein. Auch lange Dienste (42 %) sowie gesundheitspolitische Entwicklungen und Strukturveränderungen (39 %) wurden von einem großen Teil als häufig und stark belastend eingeschätzt (Abb. 3).

Ebenfalls hohe Belastungswerte zeigten sich für körperlich anstrengende Arbeit (33 % häufig belastet) sowie für psychisch belastende und aufreibende Tätigkeiten (29 % häufig, 4 % stark belastet). Damit betreffen die zentralen Belastungsfaktoren sowohl organisatorische Rahmenbedingungen als auch physische und psychische Anforderungen des klinischen Alltags.

Demgegenüber wurden interpersonelle und soziale Belastungen deutlich seltener berichtet. So gaben die meisten Befragten an, durch Mobbing (87 % gar nicht oder selten belastet) sowie durch Einsamkeit/Isolation (86 % gar nicht oder selten belastet) kaum beeinträchtigt zu sein. Auch mangelnde Unterstützung durch Kolleginnen und Kollegen (62 % gar nicht oder selten belastet) und durch Vorgesetzte (49 %) wurden überwiegend als wenig belastend wahrgenommen.

Bei der Analyse der beruflichen Belastungsfaktoren nach Tätigkeitsumfang in der endokrinen Chirurgie zeigte sich nur für die Belastung durch Nachtdienste ein signifikanter Gruppenunterschied. Die Belastung war entsprechend bei nachrangig in der endokrinen Chirurgie tätigen Befragten signifikant höher als bei überwiegend endokrin tätigen Personen (OR = 3,50; *p* = 0,003). Für alle übrigen Belastungsfaktoren ergaben sich keine signifikanten Unterschiede zwischen den Gruppen. Bei der Analyse der beruflichen Belastungsfaktoren in Abhängigkeit vom Geschlecht zeigten sich keine statistisch signifikanten Unterschiede zwischen Frauen und Männern.

**Abb. 4:**
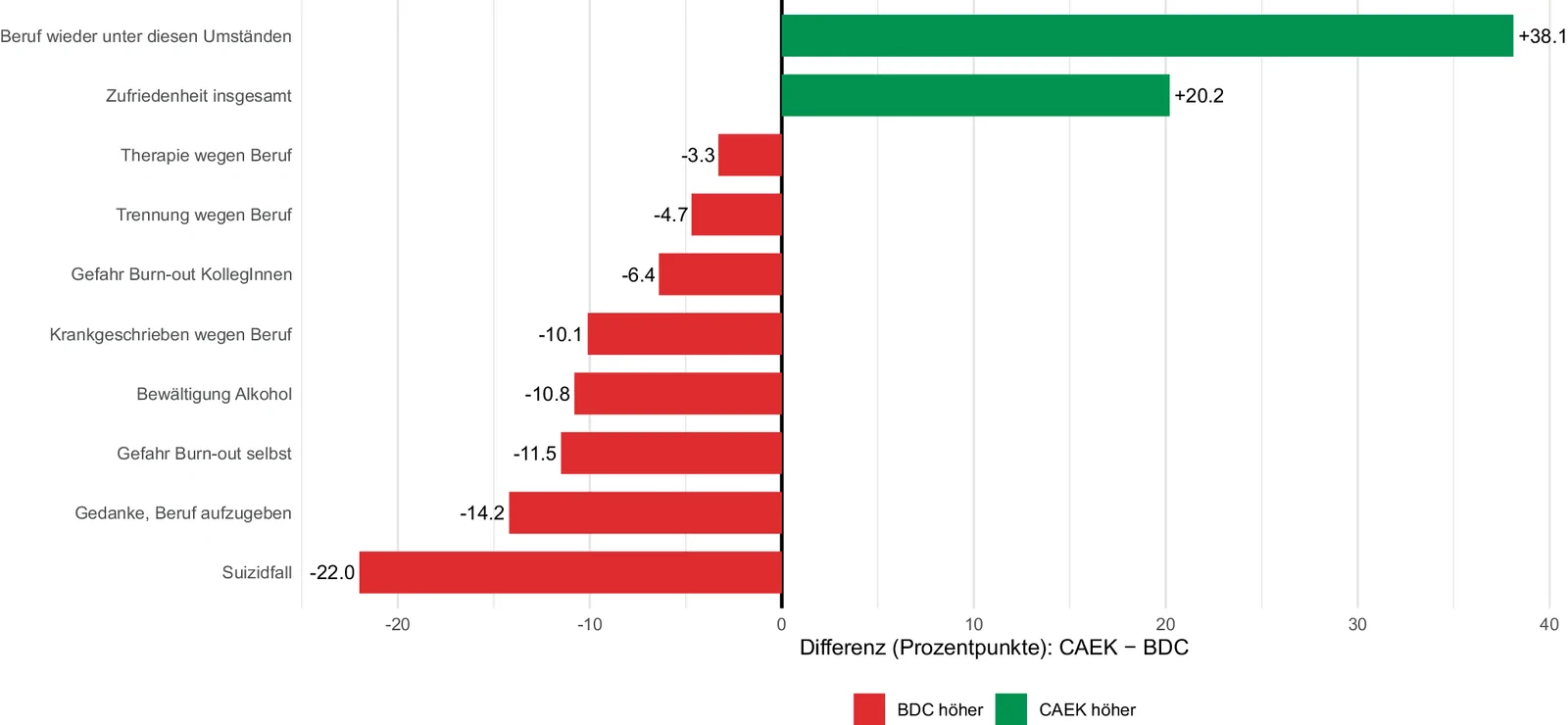
Differenzen zwischen Ergebnissen der Chirurgischen Arbeitsgemeinschaft Endokrinologie (CAEK) - und dem Berufsverband der Deutschen Chirurgie (BDC) - Umfrage in ausgewählten Zufriedenheitsindikatoren und psychosozialen Belastungsfaktoren. Dargestellt sind Differenzen in Prozentpunkten (CAEK-BDC). Positive Werte zeigen höhere Anteile in der CAEK-Gruppe, negative Werte höhere Anteile in der BDC-Gruppe

In der Gesamtstichprobe gaben 43 % der Befragten an, bereits darüber nachgedacht zu haben, den Beruf aufzugeben. Hier zeigten sich keine signifikanten Unterschiede in Abhängigkeit vom Ausmaß der Tätigkeit in der endokrinen Chirurgie. Weiterhin berichteten 8,3 %, bereits aufgrund einer schwierigen oder unerträglichen beruflichen Situation krankgeschrieben gewesen zu sein. Auch hier ergaben sich keine Unterschiede zwischen Frauen und Männern oder eine Abhängigkeit vom Ausmaß der Tätigkeit in der endokrinen Chirurgie. Bezüglich der psychischen Belastung gaben 53,6 % an, bei sich selbst bereits an die Gefahr eines Burn-outs gedacht zu haben, während 78,6 % angaben, eine solche Gefahr bei Kolleginnen oder Kollegen zu sehen. Zudem berichteten 11,9 %, aufgrund beruflicher Belastungen eine Therapie oder Beratung in Anspruch genommen zu haben; darunter Psychotherapie, Ehe- und Familienberatung, Coaching oder weitere ärztliche Konsultationen (Abb. 4).

Insgesamt gaben 67,9 % der Befragten an, dass sich die berufliche Belastung negativ auf ihre partnerschaftliche Beziehung auswirken würde. Zu einer Trennung aus beruflichen Gründen sei es bei 17,9 % der Befragten gekommen; 48,8 % gaben außerdem an, dass sich ihr Beruf negativ auf die Beziehung zu ihren Kindern auswirken würde (Abb. 4).

Als praktizierte Bewältigungsstrategien nannten jeweils 9,5 % das Rauchen bzw. den Alkoholkonsum. Medikamente oder illegale Substanzen spielten hierbei keine wesentliche Rolle.

Insgesamt 1‑mal (4,8 %) wurde von Kenntnis eines Suizidfalls oder Suizidgedanken in Zusammenhang mit beruflicher Belastung berichtet.

## Diskussion

Von den insgesamt 385 Teilnehmenden der CAEK-Tagung konnten 84 vollständig ausgefüllte Fragebögen in die Analyse einbezogen werden, was einer Rücklaufquote von etwa 22 % entspricht. Zu berücksichtigen ist dabei, dass ein Teil der Kongressteilnehmenden nicht zur primären Zielgruppe der Umfrage (in der endokrinen Chirurgie tätige Chirurginnen und Chirurgen) zählte. Unabhängig davon ist die Aussagekraft der vorliegenden Ergebnisse durch die begrenzte Stichprobengröße sowie mögliche Selektionsmechanismen eingeschränkt. Die Ergebnisse sind daher als explorativ zu interpretieren und nur eingeschränkt auf die Gesamtheit der in der endokrinen Chirurgie tätigen Ärztinnen und Ärzte übertragbar.

Im Vergleich zur bundesweiten BDC-Umfrage berichten die Teilnehmenden dieser Studie von einer insgesamt höheren beruflichen Zufriedenheit. In unserer Umfrage gaben 75 % der Befragten an, dass sie sich wieder für die endokrine Chirurgie entscheiden würden, verglichen mit nur 37 % in der BDC-Umfrage [1]. Die weiteren Differenzen der beiden Umfragen sind in Abb. 4 dargestellt. Hier zeigten sich insbesondere eine höhere Zufriedenheit in Bezug auf Fortbildungsmöglichkeiten und Arbeitszeitregelungen sowie eine geringere berichtete psychosoziale Belastung im Vergleich zur BDC-Umfrage.

Signifikant zufriedener zeigten sich die überwiegend endokrin chirurgisch tätigen Befragten in unserer Studie mit den Fortbildungsmöglichkeiten und der Weiterbildung. Ein vergleichbarer Zusammenhang zwischen chirurgischer Spezialisierung und hoher beruflicher Zufriedenheit wurde auch in anderen operativen Disziplinen beschrieben. In einer aktuellen webbasierten Befragung von Mitgliedern der *American Shoulder and Elbow Surgeons* gaben 98 % der Teilnehmenden an, mit ihrer subspezialisierten Fachwahl zufrieden zu sein und, dass sie sich trotz hoher operativer und organisatorischer Anforderungen wieder dafür entscheiden würden [6]. Diese Ergebnisse stützen die Annahme, dass das fokussierte „Eintauchen“ in ein klar definiertes chirurgisches Teilgebiet mit einer starken beruflichen Identifikation und erhöhter Zufriedenheit einhergehen kann und somit auch die Ergebnisse der vorliegenden Studie plausibel erklärt. Gleichzeitig kann eine zunehmende Spezialisierung organisatorische Herausforderungen mit sich bringen, insbesondere im Hinblick auf die Abdeckung von Hintergrunddiensten und die Integration in bestehende klinische Strukturen.

Insgesamt zeigen sich in der endokrinen Chirurgie die gleichen Hauptbelastungspunkte wie in der BDC-Umfrage: bürokratische und organisatorische Strukturen (z. B. durch Dokumentationspflichten oder administrative Prozesse). Auch im Bereich der Auswirkung auf die privaten Beziehungen (Partnerschaft, Kinder) zeigten sich mit der BDC-Umfrage vergleichbare Zahlen. Deutlich geringer war der Prozentsatz der Ärztinnen und Ärzte, die angaben, von einem Suizidfall oder Suizidabsichten im beruflichen Umfeld zu wissen (4,8 % vs. 26,8 %).

Die Aussagekraft der vorliegenden Ergebnisse ist durch mehrere Limitationen eingeschränkt. Zum einen ist ein Selection Bias nicht auszuschließen, da die Teilnehmenden im Rahmen einer Fachveranstaltung rekrutiert wurden. Dies könnte zu einer Überrepräsentation besonders engagierter oder stark mit dem Fach identifizierter Chirurginnen und Chirurgen geführt haben. Allerdings war der Zusammenhang zwischen beruflicher Zufriedenheit und dem Umfang der Tätigkeit in der endokrinen Chirurgie auch innerhalb der Teilnehmenden der CAEK nachvollziehbar, was die Aussagekraft der Ergebnisse stützt. Zum anderen schränkt die vergleichsweise geringe Zahl von 84 vollständig ausgefüllten Fragebögen die Generalisierbarkeit der Ergebnisse ein. Darüber hinaus ist zu berücksichtigen, dass im Verhältnis zur Gesamtzahl der CAEK-Mitglieder sowie zur Zahl der tatsächlich überwiegend in der endokrinen Chirurgie tätigen Ärztinnen und Ärzte nur ein begrenzter Teil der Zielgruppe erfasst wurde.

Ein weiterer wichtiger Aspekt ist die Zusammensetzung der Stichprobe: Ein Großteil der Teilnehmenden befand sich in leitenden Positionen (Oberärztinnen/Oberärzte bzw. Chefärztinnen/Chefärzte). Dies könnte die berichtete berufliche Zufriedenheit, insbesondere in Bezug auf Arbeitszeitregelungen und Fortbildungsmöglichkeiten, positiv beeinflusst haben. Gleichzeitig spiegelt diese Verteilung die strukturellen Gegebenheiten der chirurgischen Weiterbildung wider, da eine Spezialisierung in der endokrinen Chirurgie typischerweise erst in fortgeschrittenen Ausbildungsstadien erfolgt. Entsprechend waren Assistenzärztinnen und Assistenzärzte in unserer Stichprobe kaum überwiegend endokrin-chirurgisch tätig. Die Ergebnisse sind daher nur eingeschränkt auf weniger vertretene Gruppen, insbesondere Assistenzärztinnen/-ärzte, übertragbar. Eine breiter angelegte Befragung, beispielsweise unter Einbeziehung aller CAEK-Mitglieder, könnte zukünftig dazu beitragen, die vorliegenden Ergebnisse zu validieren und ein umfassenderes Bild der Arbeitsbedingungen in der endokrinen Chirurgie zu zeichnen.

Die Ergebnisse dieser Untersuchung deuten darauf hin, dass Ärztinnen und Ärzte mit überwiegender Tätigkeit in der endokrinen Chirurgie ein signifikant höheres Maß an beruflicher Zufriedenheit aufweisen, obwohl das wahrgenommene Belastungsniveau weitgehend dem ihrer Kolleginnen und Kollegen mit geringerem endokrin-chirurgischem Tätigkeitsschwerpunkt entspricht. Vor diesem Hintergrund erscheint es sinnvoll, strukturelle und organisatorische Voraussetzungen zu schaffen, die eine gezielte fachliche Schwerpunktbildung ermöglichen. Hierzu zählen insbesondere die Entwicklung geeigneter Weiterbildungsstrukturen sowie die Etablierung entsprechender klinischer Rahmenbedingungen. Eine solche strukturelle Förderung könnte langfristig dazu beitragen, die Attraktivität des Fachgebiets zu erhöhen und die Nachwuchsbindung zu stärken.

## Fazit

Die vorliegende Studie untersuchte erstmals gezielt die berufliche Zufriedenheit und die Arbeitsbedingungen in der endokrinen Chirurgie. Trotz vergleichbarer Gesamtbelastung zeigten Ärztinnen und Ärzte mit überwiegend endokrin-chirurgischem Schwerpunkt eine signifikant höhere Zufriedenheit (OR = 2,78); 75 % würden sich uneingeschränkt erneut für das Fach entscheiden. Entscheidend hierfür waren strukturelle und tätigkeitsbezogene Faktoren, insbesondere Fortbildung, Arbeitszeitregelung und Weiterbildung, während Bürokratie und organisatorische Anforderungen die Hauptbelastung darstellten. Die Ergebnisse legen nahe, dass eine klare fachliche Profilbildung und strukturierte Weiterbildungswege die Attraktivität hoch spezialisierter Tätigkeitsprofile fördern. Geeignete strukturelle Rahmenbedingungen erscheinen somit als zentraler Hebel, um Arbeitszufriedenheit, Nachwuchsbindung und eine nachhaltige chirurgische Versorgung langfristig zu sichern.

## Data Availability

Die erhobenen Datensätze können auf begründete Anfrage in anonymisierter Form beim korrespondierenden Autor angefordert werden. Die Daten befinden sich auf einem Datenspeicher am TUM Klinikum rechts der Isar.
